# Upconverting Nanoparticles for Bimodal Luminescence and Magnetic Resonance Imaging of Langerhans Islets

**DOI:** 10.1002/smsc.70344

**Published:** 2026-07-16

**Authors:** Oleksandr Shapoval, Daniel Jirák, Zuzana Berková, Miroslav Šlouf, Olga Kočková, Hana Engstová, Aminadav Halili, David Červený, Petr Ježek, Daniel Horák

**Affiliations:** ^1^ Institute of Macromolecular Chemistry of the Czech Academy of Sciences Prague Czech Republic; ^2^ Institute for Clinical and Experimental Medicine Prague Czech Republic; ^3^ Institute of Biophysics and Informatics First Faculty of Medicine Charles University Prague Czech Republic; ^4^ Faculty of Health Studies Technical University of Liberec Liberec Czech Republic; ^5^ Institute of Physiology of the Czech Academy of Sciences Prague Czech Republic

**Keywords:** bimodal imaging, Langerhans islets, luminescence, MRI, upconverting nanoparticles

## Abstract

Combining luminescence with magnetic resonance imaging (MRI) is a noninvasive approach that significantly improves detection sensitivity and diagnostic precision for severe diseases. In diabetes care, this approach considerably broadens the possibilities for monitoring of Langerhans islet transplantation by improving their detectability and quantification within existing imaging modalities, including MRI. To realize this concept, upconverting nanoparticles (UCNPs) appear particularly promising, providing bimodal MRI and luminescence with the ability of near‐infrared (NIR) light to penetrate deep into tissues. In this work, novel monodisperse dumbbell‐shaped core–shell UCNPs (CS‐UCNPs) coated with poly(methyl vinyl ether‐*alt*‐maleic acid) (PMVEMA) are developed for the bimodal imaging of Langerhans islets. Codoping of Fe, Yb, and Er ions in the NaYF_4_ host matrix, along with the presence of NaGdF_4_:Nd, Yb, Tb shell, increases both *r*_1_ and *r*_2_ relaxivities and upconversion luminescence in the red region, which is suitable for in vivo applications. The biocompatible PMVEMA coating ensures colloidal stability of the particles in aqueous physiological fluids and their nontoxicity. The potential of CS‐UCNPs for simultaneous MRI and optical visualization is tested on isolated Langerhans islets. The efficiency of in vivo visualization of CS‐UCNP@PMVEMA‐labeled Langerhans islets transplanted under the kidney capsule in a rat model is investigated using *T*_1_‐, *T*_2_‐, and *T*_2_*‐weighted MRI sequences.

## Graphical abstract

Monodisperse dumbbell‐shaped core–shell upconversion nanoparticles (CS‐UCNPs) codoped with Fe, Yb, Er ions and coated with NaGdF_4_:Nd, Yb, Tb shell provide efficient *T*_1_/*T*_2_^*^‐weighted magnetic resonance imaging (MRI) contrast and bright red emission under near‐infrared excitation. Colloidally stable poly(methyl vinyl ether‐*alt*‐maleic acid) (PMVEMA)‐coated CS‐UCNPs enable simultaneous MRI in vivo tracking of transplanted islets in a rat combined with optical verification of nanoparticle labeling in isolated Langerhans islets. Schematic illustration of the design of upconverting nanoparticles for bimodal luminescence and magnetic resonance imaging of Langerhans islets.

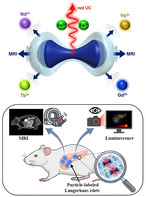

## 1 Introduction

Diabetes mellitus, characterized by chronic hyperglycemia and progressive organ dysfunction, is an increasingly prevalent life‐threatening disease worldwide [1]. Early detection and monitoring of this disease progression are crucial for advancing effective therapeutic treatments [2], such as Langerhans (pancreatic) islet transplantation [3]. However, several significant imaging challenges arise due to the deep anatomical location of the pancreas, the small size of islets (50–600 µm), and their dispersed distribution (1%–3% of gland volume) within predominantly exocrine tissue [4]. Direct in vivo visualization of native or unlabeled transplanted Langerhans islets has so far been unsuccessful, mainly due to the low affinity of contrast agents for β‐cells, minimal tissue contrast, and relatively low islet density within the pancreas or when transplanted in liver [5, 6]. Moreover, the narrow interstices between the cells in the islets hinder the action of immunotherapeutic approaches. An alternative strategy involves labeling of pancreatic islets in vitro before transplantation to enable subsequent visualization, but their accurate quantification remains challenging [7, 9].

To overcome these limitations, multimodal imaging techniques combining optical luminescence with magnetic resonance imaging (MRI) have been explored [10, 14]. These techniques offer complementary advantages, notably improved sensitivity, high resolution, and deep tissue penetration. MRI provides excellent soft tissue contrast without ionizing radiation, although it may be contraindicated in patients with certain metallic implants. Optical luminescence supports intraoperative guidance and preclinical in vivo applications, enabling subcellular resolution for ex vivo imaging [15]. However, its application is often limited by photobleaching, rapid signal decay, and poor penetration depth. In contrast, upconversion luminescence, which converts near‐infrared (NIR) light to UV and/or visible light, prevents light scattering and tissue autofluorescence, while NIR radiation allows relatively deep tissue penetration [16]. This enables detection of low abundance disease markers for personalized medicine and bioanalytical applications [17]. Combining upconversion luminescence with other imaging modalities offers great benefits for both clinical diagnostics and biomedical research, allowing a deeper understanding of treatment efficacy, including in vivo targeted drug delivery for diabetes and noninvasive visualization of functional β‐cells [18, 19].

Over the past decade, rapid development of nanotechnology has led to new opportunities for improving the efficiency of existing noninvasive visualization methods. Lanthanide‐based upconverting nanoparticles (UCNPs) show strong potential for accurate clinical imaging [20]. The optical properties of UCNPs are enabled by the unique characteristics of lanthanide ions, such as anti‐Stokes luminescence and stable imaging during long‐term observation. Their paramagnetic properties enable interaction with water protons to produce MRI “hypersignal” in tissues [10]. The combination of luminescent and magnetic properties of UCNPs facilitates high‐resolution imaging, advancing both clinical diagnostics and biomedical research [21].

To date, magnetic iron oxide nanoparticles have enabled in vivo visualization of pancreatic β‐cells and islets due to favorable biocompatibility, high spatial resolution, and the potential for long‐term monitoring [22, 25]. Other contrast agents, including radiolabeled exendin [26], zinc [27], fluorine [19, 28], gadolinium [29, 30], manganese [31], silver [32], and gold, have also been suggested for islet and β‐cell imaging [33]. Their main disadvantages include toxicity, low in vivo sensitivity, and specificity because of poor correlation between the particle concentration and ^1^H magnetic resonance signal [34]. Considering these current limitations of single‐modal imaging, a robust diagnostic system is needed that allows multimodal visualization of pancreatic islets and β‐cells without affecting their functionality.

Recently, we developed poly(4‐styrenesulfonic acid‐*co*‐maleic anhydride)‐coated NaGdF_4_:Yb,Tb,Nd nanoparticles for multimodal imaging of pancreatic Langerhans islets and β‐cells, although this coating did not provide optimal toxicity and stability of the particles in physiological fluids [14]. In this report, we focused on the design of new poly(methyl vinyl ether‐*alt*‐maleic acid) (PMVEMA)‐coated dumbbell‐shaped NaYF_4_:Yb,Er,Fe@NaGdF_4_:Nd,Yb,Tb UCNPs that combine upconversion luminescence with MRI detection and on the assessment of their potential for Langerhans islet imaging.

## 2 Experimental

### 2.1 Materials

Sodium, ytterbium(III), yttrium(III), terbium(III), erbium(III), neodymium(III), gadolinium(III), and iron(II) chlorides; phosphate‐buffered saline (PBS; pH = 7.4); ammonium fluoride; octadec‐1‐ene; oleic acid; fetal bovine serum (FBS; 10%); 5% *N*‐2‐hydroxyethylpiperazine‐*N*′‐2‐ethanesulfonic acid (HEPES buffer); 1% penicillin/streptomycin solution; 1% Glutamax; Ficoll solutionl Alamar Blue test; propidium iodide; acridine orange; and collagenase were purchased from Merck (Darmstadt, Germany). Ethanol, hexane, sodium hydroxide, and methanol were obtained from Lach‐Ner (Neratovice, Czech Republic). Poly(methyl vinyl ether‐*alt*‐maleic acid) (PMVEMA; *M*_w_ = 60 kDa) was obtained from Scientific Polymer Products (Ontario, NY, USA). Distilled demineralized water (conductivity <0.1 µS/cm) filtered on a Milli‐Q Gradient A10 system (Millipore; Molsheim, France) was used during all experiments. Other reagent grade chemicals were obtained from commercial sources and used as received.

### 2.2 Synthesis of Core‐ and Core–Shell UCNPs and Their Surface Modification With PMVEMA

Core NaYF_4_:Yb,Er,Fe nanoparticles (C‐UCNPs) were synthesized similarly as described in our previous publication with some modifications [35]. Briefly, in a 100‐mL round‐bottom three‐neck flask, a mixture of metal chlorides (Y^3+^/Yb^3+^/Er^3+^/Fe^2+^ = 0.55:0.2:0.15:0.1 mmol/mmol/mmol/mmol) in oleic acid (6 mL) was added to 15 mL of octadec‐1‐ene and heated to 170 °C for 30 min with magnetic stirring in an argon atmosphere to obtain a homogeneous solution of oleates. After cooling to room temperature (RT), a solution of NaOH (2.5 mmol) and NH_4_F (4 mmol) in methanol (10 mL) was added, methanol was evaporated, and the mixture was slowly heated to 300 °C for 90 min with stirring under an argon atmosphere. The resulting C‐UCNPs were separated by centrifugation (3460 rcf) for 30 min, washed with hexane and ethanol 3 times, and dispersed in hexane.

The core–shell NaYF_4_:Yb,Er,Fe@NaGdF_4_ (CS‐UCNPs I) and NaYF_4_:Yb,Er,Fe@NaGdF_4_:Yb,Tb,Nd nanoparticles (CS‐UCNPs II) were prepared according to the procedure described earlier [36]. Briefly, lanthanide oleates were obtained by dissolving of lanthanide chlorides (Gd^3+^/Nd^3+^/Yb^3+^/Tb^3+^ = 0.45/0.05/0.05/0.05 mmol/mmol/mmol/mmol) in 6 mL of oleic acid and 15 mL of octadec‐1‐ene, which was followed by heating at 160 °C for 30 min under an argon atmosphere. Optionally, gadolinium oleate (0.6 mmol Gd^3+^) was used for the synthesis of an undoped NaGdF_4_ shell. After cooling to RT, a hexane dispersion (15 mL) of C‐UCNPs (150 mg) and methanolic solution of NaOH (1.25 mmol) and NH_4_F (2 mmol) were added. The mixture was slowly heated to 120 °C until methanol and hexane evaporated and the reaction continued at 300 °C for 1.5 h under an argon atmosphere. The CS‐UCNPs were separated by centrifugation (3460 rcf) for 30 min and washed with hexane/ethanol mixture 4 times to remove oleic acid. Finally, the particles were washed with water/ethanol solution 3 times, gradually replacing ethanol with aqueous ethanol, and redispersed in water. A part of the particle dispersion was dried in a vacuum at RT for 3 days and physicochemical characterized. The surface modification of CS‐UCNPs II with PMVEMA was performed according to our earlier report and the resulting particles were denoted as CS‐UCNP@PMVEMA [37].

### 2.3 Characterization Methods

Nanoparticle morphology, elemental composition, and crystal structure were analyzed using a Tecnai Spirit G2 transmission electron microscope (TEM; FEI; Brno, Czech Republic) equipped with energy‐dispersive spectrometer (EDX) and selected‐area electron diffraction (SAED). Samples for microscopic characterization were prepared as follows: 3 μL of the nanoparticle dispersion was dropped onto a standard TEM copper grid covered with an ultrathin electron transparent carbon film, excess liquid was removed with filter paper, and the sample was left to dry at ambient temperature. The grids were inserted in the microscope and observed at the accelerating voltage of 120 kV using standard bright‐field imaging (TEM/BF). The average particle size and particle size distribution were determined by measuring at least 250 nanoparticles from TEM micrographs using ImageJ2 software version 1.52p (National Institutes of Health; Bethesda, MD, USA) [38]. For each particle type, standard morphological equivalent diameter of *i‐*th particle (denoted as *D* *_i_* in Equations ((1) ‐ (3)), *MinFeret* (particle width) and *MaxFeret* (particle length) were determined; their definitions can be found in ImageJ2 documentation [38]. Number‐average particle diameter (*D*_n_), weight‐average particle diameter (*D*_w_), and the uniformity (dispersity *Ð*) were calculated as follows [39]:

(1) $$D_{n}=\sum N_{i}\cdot D_{i}/\sum N_{i}$$

(2) $$D_{w}=\sum N_{i}\cdot {D_{i}}^{4}/\sum N_{i}\cdot {D_{i}}^{3}$$

(3) $$\text{Ð}=D_{w}/D_{n}$$

where *N* *_i_* is the number of nanoparticles with diameter *D* *_i_*. Elemental composition was verified by energy‐dispersive spectroscopy (TEM/EDX; detector EDAX, Mahwah, NJ, USA). Crystal structure was determined from selected‐area electron diffraction (TEM/SAED). The TEM/SAED patterns were converted to 1D‐profiles and compared with theoretically calculated powder X‐ray diffraction (PXRD) patterns of cubic (α) and hexagonal (β) sodium yttrium fluoride; the complete processing was made with program package EDIFF as described elsewhere [40, 41].

PXRD patterns were measured using Bragg–Brentano geometry under the Cu Kα radiation (wavelength *λ* = 1.54 Å) using a high‐resolution GNR Explorer diffractometer (Novara, Italy) with a Mythen 1K strip detector, in the 2*θ* range of 10° – 80° with a step of 0.1° and a time of 15 s at each step. The degree of crystallinity was calculated from the ratio of crystalline region’s area to the total area in the XRD pattern. The experimental PXRD patterns were compared with the theoretically calculated patterns of NaYF_4_ and NaGdF_4_ crystals to estimate the structural changes as a function of gadolinium concentration. The calculations were performed with program package EDIFF [41].

The hydrodynamic diameter (*D*_h_), ζ‐potential, and polydispersity (*PD*) of nanoparticles were determined by dynamic light scattering (DLS) at 25 °C using a ZSU 5700 Zetasizer Ultra instrument (Malvern Instruments; Malvern, UK); *D*_h_ and *PD* were calculated from the intensity‐weighted distribution function obtained by CONTIN analysis of the correlation function embedded in Malvern software.

The content of lanthanide (Er^3+^, Gd^3+^, Tb^3+^, Yb^3+^, and Nd^3+^) and iron ions was quantified by inductively coupled plasma mass spectrometry (ICP‐MS) using a NexION 2000B apparatus (PerkinElmer; Waltham, MA, USA) after the digestion of particles with HNO_3_ using a Biotage initiator microwave reactor (Uppsala, Sweden). Standard solutions of Yb, Er, Gd, Tb, Nd, and Fe (100 mg/L) in 5% HNO_3_ diluted to a concentration of 0.002–0.18 μg/L were used to obtain a calibration curve. The metal ions in the samples were stabilized by the addition of 2.5% HNO_3_. The measurements were performed with a collision cell (He mode) to successfully eliminate polyatomic interferences (such as ^40^Ar^16^O^+^).

Thermogravimetric analysis (TGA) of particles was performed in air with a Perkin Elmer TGA 7 analyzer (Norwalk, CT, USA) over the temperature range 30–700 °C at a constant heating rate of 5 °C/min. Infrared spectra of the dried powdered samples in KBr pellets were recorded on a Nexus Nicolet 870 FTIR spectrometer (Madison, WI, USA) using a MKII Golden Gate single attenuated total reflection (ATR‐FTIR; Specac; Orpington, Kent, UK) with deuterated triglycine sulfate (DTGS) detector.

Luminescence emission and excitation spectra were recorded at RT on a FS5 spectrofluorometer (Edinburgh Instruments; Edinburgh, UK) equipped with 808 (MDL‐III‐808) and 980 nm (MDL‐III‐980) continuous wave infrared diode lasers, each with 2 W output power (beam size of 5 × 8 mm^2^).

### 2.4 Longitudinal (*T*_1_) and Transverse Relaxation (*T*_2_) and Relaxivity Measurement

*T*_1_ and *T*_2_ relaxation times of phantoms containing different particle concentrations were measured on a 1.5 T Minispec MQ60 relaxometer (Bruker; Ettlingen, Germany) at 37 °C. The *T*_1_ relaxation times were determined using an inversion recovery pulse sequence with a repetition time from 0.01 to 10,000 ms, a recycle delay 4 s, and four signal averages; data were fitted to a monoexponential model using 10 acquisition points. The *T*_2_ relaxation times were measured with the Carr–Purcell–Meiboom–Gill (CPMG) pulse sequence, echo time 0.05 ms, recycle delay 2 s, and eight signal averages; a monoexponential fit was performed based on 20,000 data points. Each sample was measured 3 times under identical conditions. Distilled water served as a reference. After subtracting the water contribution, *R*_1_ and *R*_2_ relaxation rates (s^–1^) versus actual metal ion concentration (10^–3^ M) were converted to *r*_1_ and *r*_2_ relaxivities, respectively.

### 2.5 Cell Viability Assay

Cytotoxicity of CS‐UCNP@PMVEMA nanoparticles was determined by the Alamar Blue assay. Briefly, murine mammary carcinoma 4T1 cells (ATCC; Manassas, VA, USA) were cultured in Gibco advanced RPMI medium (Thermo Fisher Scientific; Waltham, MA, USA) supplemented with 10% (v/v) FBS and 1% penicillin–streptomycin (Biosera; Cholet, France) at 37 °C for 24 h under humidified 5% CO_2_ atmosphere. Cells were seeded in 96‐well plates at a concentration of 5 × 10^4^ cells per well in complete growth medium for 24 h and then incubated with the nanoparticles (0.1–0.3 mg/mL) for 24 and 48 h under the same atmosphere. After that, the cells were washed with PBS 3 times and 10% Alamar Blue solution in culture medium was added and incubation continued for 4 h. In vitro cell viability was determined by Alamar Blue test using an Infinite 200 PRO reader (Tecan; Männedorf, Switzerland) measuring an absorbance at 570 and 600 nm. Untreated cells in RPMI medium served as the positive control. Each experiment was performed in triplicate (*n* = 3) and repeated 3 times.

### 2.6 Isolation, Labeling, and Vitality of the Langerhans Islets

Pancreatic Langerhans islets were isolated from adult male Wistar‐Kyoto (IKEM; Prague, Czech Republic) and Lewis rats (Charles River Laboratories; Sulzfeld, Germany) according to a standard isolation protocol [42]. Briefly, pancreases were perfused with collagenase solution (1 mg/mL) and digested at 37 °C. To remove the exocrine tissue, the islets were separated by Ficoll density gradient centrifugation (680 rcf) and incubated for 20 h with CS‐UCNP@PMVEMA nanoparticles (2 mg/mL) in CMRL‐1066 culture medium (PAN‐Biotech; Aidenbach, Germany) supplemented with 10% FBS, 5% HEPES buffer, 1% penicillin/streptomycin solution and 1% Glutamax in humidified incubator at 37 °C and 5% CO_2_ atmosphere. Labeled islets were hand‐picked and counted for the preparation of gelatin phantoms, vitality testing, and transplantation experiments. All experiments were ethically reviewed and performed under the European Directive 86/609/EEC and approved by the Experimental Animals Welfare Committee of the Institute for Clinical and Experimental Medicine and the Ministry of Health of the Czech Republic (No. 3/2024).

Islet vitality expressed as a percentage of live cells obtained from adult male Wistar–Kyoto rats was assessed as the difference between dead (red) and live cells (green) after staining with propidium iodide and acridine orange. The results were quantified as mean ±SD and Mann–Whitney test was used for statistical analysis.

Gelatin phantoms were prepared by placing islets from Wistar–Kyoto rats between two layers of gel (4% on the bottom layer and 2% on the top layer). Sample groups were placed in separate corners of each phantom: ∼10, ∼100, and ∼1000 pancreatic Langerhans islets labeled with CS‐UCNP@PMVEMA particles along with corresponding unlabeled controls, each located in separate corners of each phantom. This configuration enabled direct comparison of samples and efficient evaluation of MRI properties for the CS‐UCNP@PMVEMA‐labeled versus unlabeled islets under identical imaging conditions.

### 2.7 In Vitro MRI of Phantoms Containing Langerhans Islets

To assess MRI contrast properties and signal detectability of CS‐UCNP@PMVEMA nanoparticles, all samples were initially measured in phantoms to evaluate their signal detection efficiency. MRI of phantoms containing Langerhans islets was performed on a 7 T Bruker Biospec 70/30 MR spectrometer equipped with surface multiple receiver radiofrequency (Rx RF) coils with a diameter of 20 mm in combination with a circularly polarized transmitter/receiver TxRx RF coil with diameter of 98 mm. *T*_1_ *‐*, *T*_2_‐, and *T*_2_*‐weighted MRI were taken using multispin rapid acquisition with relaxation enhancement (RARE) and gradient echo fast low angle shot (FLASH) sequences. Postprocessing and signal quantification were conducted using the ImageJ software version 1.52p. Signal‐to‐noise ratio (SNR) and contrast‐to‐noise ratio (CNR) were calculated as follows from Equations (4) and (5):

(4) $$\text{SNR =}\;0.655\text{⋅}S_{s}/\sigma$$

(5) $$\text{CNR =}\;0.655\text{⋅}\left(S_{s}‐S_{w}\right)/\sigma$$

where 0.655 is a correction factor reflecting the Rician distribution of noise in magnitude MRI, *S*_w_ and *S*_s_ denote the signal intensity of water and the sample region, respectively, and *σ* is the standard deviation of background noise. The *T*_1_ and *T*_2_ relaxation times were measured with inversion recovery and CPMG sequences, respectively; parameters are shown in the Supporting Information (Table S1).

### 2.8 In Vitro Confocal Microscopy of Langerhans Islets

The CS‐UCNP@PMVEMA‐labeled Langerhans islets placed on coverslips were imaged with a Leica SP8 confocal microscope (Leica Microsystems; Wetzlar, Germany) using a 405 nm lamp in bright‐field mode and a Coherent 170 fs pulsed Chameleon laser at 980 nm (70 mW) and 808 nm (160 mW) excitation.

### 2.9 In Vivo MRI of Transplanted Langerhans Islets

Five hundred Langerhans islets obtained from a Lewis rat and labeled with CS‐UCNP@PMVEMA nanoparticles (2.3 mg/mL) were transplanted in nondiabetic Lewis rats under the renal capsule with a site selected based on favorable anatomical characteristics under general anesthesia using 5% isoflurane for induction and 1.5%–2% for maintenance. *T*_2_*‐weighted MRI was performed immediately after surgery and 3 weeks after the procedure. Identical imaging sequences and parameters to those used in the phantom experiments were employed at both time points, enabling direct comparison and assessment of signal retention, particle stability in biological tissues, and longitudinal noninvasive monitoring of the transplanted islets. After the second in vivo scan, the kidney was surgically explanted for visual inspection and high‐resolution *T*_1_‐weighted MRI. *T*_1_‐ and *T*_2_*‐weighted gradient echo MRI used the following settings: echo time 10 ms, repetition time 200 ms, flip angle 90°, a spatial resolution 0.313 × 0.313 mm, scan time 7 min for *T*_2_*‐weighted MRI of the rat and 15 min, repetition time 90 ms, echo time 5 ms, and flip angle 30° for *T*_1_‐weighted MRI of the explanted kidney. All in vivo experiments were designed as pilot proof‐of‐concept studies in accordance with the 3R principles to demonstrate the feasibility of MRI‐based visualization of CS‐UCNP@PMVEMA‐labeled islets, without aiming at inferential statistical comparison.

## 3 Results and Discussion

### 3.1 Synthesis and Characterization of UCNPs

In this study, a high‐temperature coprecipitation method was used to synthesize NaYF_4_:Yb,Er,Fe core (C‐UCNPs) and NaYF_4_:Yb,Er,Fe@NaGdF_4_ (CS‐UCNPs I) and NaYF_4_:Yb,Er,Fe@NaGdF_4_:Yb,Tb,Nd core–shell nanoparticles (CS‐UCNPs II). While commonly used NaYF_4_:Yb,Er C‐UCNPs were characterized by strong green emission and negligible red emission, the incorporation of Fe^2+^ ions into the particles increased luminescence intensity in the red region, making them more suitable for in vivo applications [43, 44]. The Gd‐, Tb‐, Yb‐, and Nd‐containing shell introduced around the C‐UCNPs enhanced MRI contrast and enabled excitation in the transparent NIR optical window at 808 nm characterized by deep penetration into biological tissues [14, 45, 46]. It is a biologically more favorable NIR wavelength than 980 nm because of its lower water absorption and reduced risk of tissue heating. In addition, the shell increased upconversion emission by passivating the optically active ions on the cores, thereby preventing surface quenching.

**FIGURE 1:**
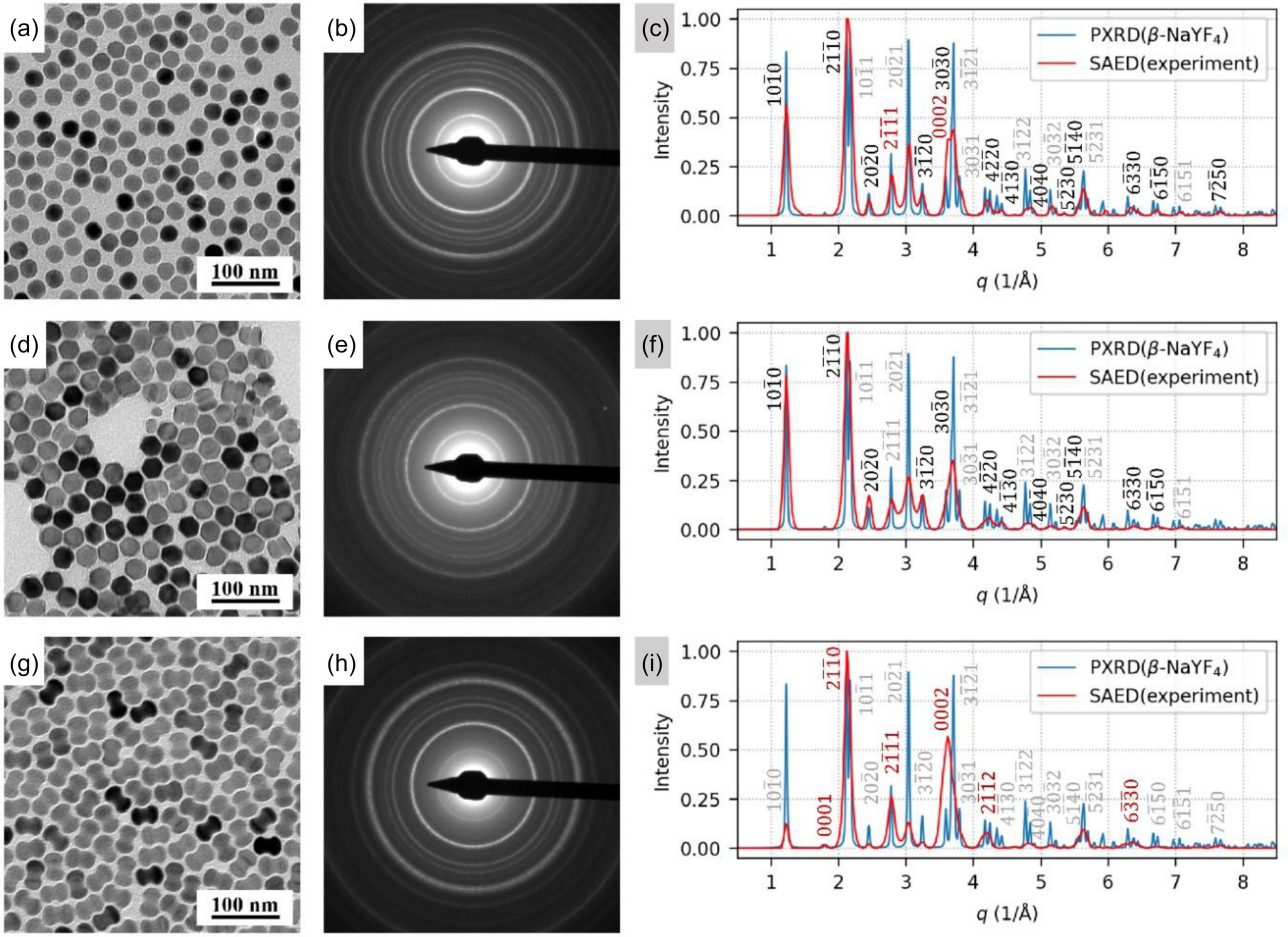
(a,d,g) TEM/BF micrographs and (b,e,h) experimental TEM/SAED diffraction patterns of (a–c) C‐UCNPs, (d–f) CS‐UCNPs I, and (g–i) CS‐UCNPs II nanoparticles; (c,f,i) comparison of the radially averaged TEM/SAED diffraction patterns (red lines) with the theoretically calculated PXRD patterns of hexagonal β‐NaYF_4_ (blue lines). The Weber indices (*uvtw*) in the diffractograms were marked with different colors corresponding to different preferred orientations of the nanocrystals: black—preferred orientation with zone axis [*hkl*] = [001], dark red—preferred orientation with zone axis [*hkl*] = [120], and gray—other orientations.

**FIGURE 2:**
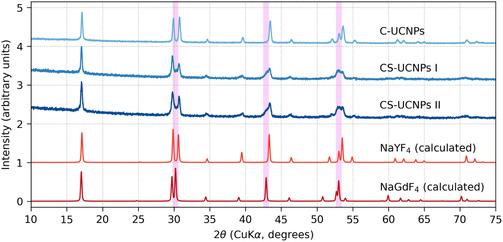
Experimental PXRD pattern of C‐UCNPs (light blue), CS‐UCNPs I (medium blue), and CS‐UCNPs II nanoparticles (dark blue) compared to the theoretically calculated PXRD patterns of β‐NaYF_4_ (orange; JCPDS card no. 28‐1192) and β‐NaGdF_4_ phase (red; JCPDS card no. 27‐0699).

The morphology, crystal structure, and elemental composition of C‐ and CS‐UCNPs were analyzed by TEM (Table 1; Figures 1, 2 and S1). C‐UCNPs (Figure 1a–c), CS‐UCNPs I (Figure 1d–f), and CS‐UCNPs II (Figure 1g–i) exhibited almost monodisperse size distributions with different morphology and crystal structures. Particle size increased gradually from C‐UCNPs through CS‐UCNPs I to CS‐UCNPs II (Table 1; Figure S1). C‐UCNPs were spherical with mean diameter *D*_*n*_ = 26 nm and monodisperse size distribution (*Ð* = 1.01; Table 1; Figures 1a and S1). After introducing undoped NaGdF_4_ shell on the cores, the size of CS‐UCNPs I increased to 31 nm and the shape changed from isometric to predominantly hexagonal (*Ð* = 1.06; Table 1; Figures 1d and S1). Interestingly, after the introduction of NaGdF_4_:Yb,Tb,Nd shell around the NaYF_4_:Yb,Er,Fe core, the CS‐UCNPs II had a uniform dumbbell‐shaped morphology with a width of ∼27 nm and a length of ∼42 nm and monodisperse size distribution (*D*_*n*_ = 35 nm; *Ð* = 1.00; Table 1; Figures 1g and S1). The narrow size distribution is critical for consistent physical and biological characteristics ensuring the high quality of the synthesized particles and reproducibility of the results [47]. Analogous dumbbell‐shaped morphologies were reported for NaErF_4_:Yb,Tm and NaYbF_4_:Er,Tm UCNPs after the deposition of both NaYF_4_:Yb inner and NaNdF_4_:Yb outer shells [48, 49]. The dumbbell‐shaped morphology of CS‐UCNPs II was primarily due to the lattice mismatch between the NaYF_4_:Yb,Er,Fe core and the NaGdF_4_:Yb,Tb,Nd shell. The resulting strain caused directional (anisotropic) growth of the shell at the ends of the core particles. Additionally, the spatial distribution of Tb^3+^, Yb^3+^, and Nd^3+^ ions could modify the local chemical environment and affect the nucleation and growth rates at different edges of the nanoparticle [50].

**TABLE 1:** TEM and DLS characterization of UCNPs.

Particles	*D*_n_, nm	*Ð*	*D*_h_, nm	*PD*	ζ‐potential, mV
C‐UCNPs	26 ± 1	1.01	105 ± 0.5	0.15	30 ± 2
CS‐UCNPs I	31 ± 4	1.06	175 ± 4	0.21	23 ± 1
CS‐UCNPs II	35 ± 1 (27^a^–42^b^)	1.00	188 ± 4	0.25	31 ± 3
CS‐UCNP@PMVEMA			149 ± 2	0.16	−28 ± 2

*Notes:* C‐UCNPs, CS‐UCNPs I, and CS‐UCNPs II—core NaYF_4_:Yb,Er,Fe and core–shell NaYF_4_:Yb,Er,Fe@NaGdF_4_ and NaYF_4_:Yb,Er,Fe@NaGdF_4_:Yb,Tb,Nd upconverting nanoparticles, respectively; *D*_n_—number‐average diameter (TEM); *Ð*—dispersity (TEM); *D*_h_—hydrodynamic diameter (DLS); *PD*—polydispersity (DLS).

a Width.

b Length.

The crystal structures of synthesized C‐ and CS‐UCNPs corresponded to hexagonal β‐NaYF_4_ and/or hexagonal β‐NaGdF_4_. The difference between diffraction patterns of isostructural β‐NaYF_4_ and β‐NaGdF_4_ was too low to be resolved by electron diffraction, but the presence of β‐NaGdF_4_ was proved by PXRD, as discussed below. Differences among experimental diffraction patterns (Figure 1b,e,h) were associated with different particle morphology resulting from various preferred orientations of the nanocrystals deposited on the supporting carbon film [51]. The thin flat hexagonal CS‐UCNPs I nanocrystals lay on their hexagonal bases with the (*hkl*) = (001) crystallographic plane oriented parallel to the substrate (Figure 1d). The normal to the (*hkl*) = (001) plane was the [*uvw*] = [001] direction corresponding to zone axis [52]. Applying the Weiss zone law (WZL; *hu* + *kv* + *lw* = 0) to zone axis [*uvw*] = (001) showed that the indices of the strongest diffractions have to satisfy the condition *h⋅*0 + *k⋅*0 + *l⋅*1 = 0, i.e., *l* = 0. This was in agreement with the strongest TEM/SAED diffraction patterns of CS‐UCNPs I with the final index of *l* = 0 (Figure 1f; red curve marked with black font). It should be noted that EDIFF software used four‐number Weber indices (*hkil*), to which an additional index (*i = –h+k*) was added for hexagonal lattices [53]. This extra index did not affect the final results and the all above‐listed conclusions for (*hkl*) indices remained valid. The elongated dumbbell‐shaped CS‐UCNPs II nanocrystals had the same crystal structure as CS‐UCNPs I, but a different orientation (Figure 1g). They mostly laid on the lateral faces of the deformed dumbbell‐shaped hexagonal prisms, which corresponded to the (*hkl*) = (100) planes parallel to the substrate, whose normal directions were [*uvw*] = [120]. Application of WZL to the zone axis [*uvw*] = [120] led to the condition for the indices of the strongest diffractions *h*⋅1 + *k*⋅2 + *l*⋅0 = 0, i.e., *h* = −2*k*. The experimental TEM/SAED diffractogram of CS‐UCNPs II confirmed the above considerations, as the indices of all strong diffractions satisfied the condition *h* = −2*k* (Figure 1i; red curve marked with red font). The diffraction pattern of the initial isometric C‐UCNPs showed a mixture of two preferred orientations (Figure 1c; red line). The first preferred orientation corresponded to the zone axis [*uvw*] = [001] due to the strongest diffractions of CS‐UCNPs I with *l* = 0 (Figure 1c; black font). The second preferred orientation corresponded to the zone axis [*uvw*] = [100], similar to the strongest diffractions of CS‐UCNPs II with *h* = −2*k* (Figure 1c; red font). In summary, the electron diffraction confirmed that all prepared nanocrystals exhibited the crystal structure of hexagonal β‐NaYF_4_ (or the isostructural β‐NaGdF_4_) and the differences in their diffraction patterns could be attributed to various preferred orientations of the nanocrystals on the substrate.

The phase composition and crystal structure of C‐ and CS‐UCNPs were also examined by PXRD (Figure 2). Due to the higher resolution of XRD in comparison with electron diffraction, the PXRD measurements enabled to differentiate between the isostructural β‐NaYF_4_ and hexagonal β‐NaGdF_4_ phases. The PXRD patterns of C‐UCNPs demonstrated sharp and intense peaks, indicating a well‐ordered crystalline structure. All peaks were indexed to a standard hexagonal phase of β‐NaYF_4_ (JCPDS card no. 28‐1192) with the high degree of crystallinity (79%). The XRD patterns of CS‐UCNPs I and II displayed several additional weak 2*θ* peaks at 30.25°, 30.42°, 43.03°, and 60.10° corresponding to a hexagonal β‐NaGdF_4_ phase (JCPDS card no. 27‐0699) with the degree of crystallinity 71% and 69%, respectively. The lower lattice symmetry of the hexagonal β phase (size > 20 nm) made radiative transitions more efficient and reduced nonradiative losses, resulting in enhanced upconversion luminescence compared with the cubic α‐phase [54].

**FIGURE 3:**
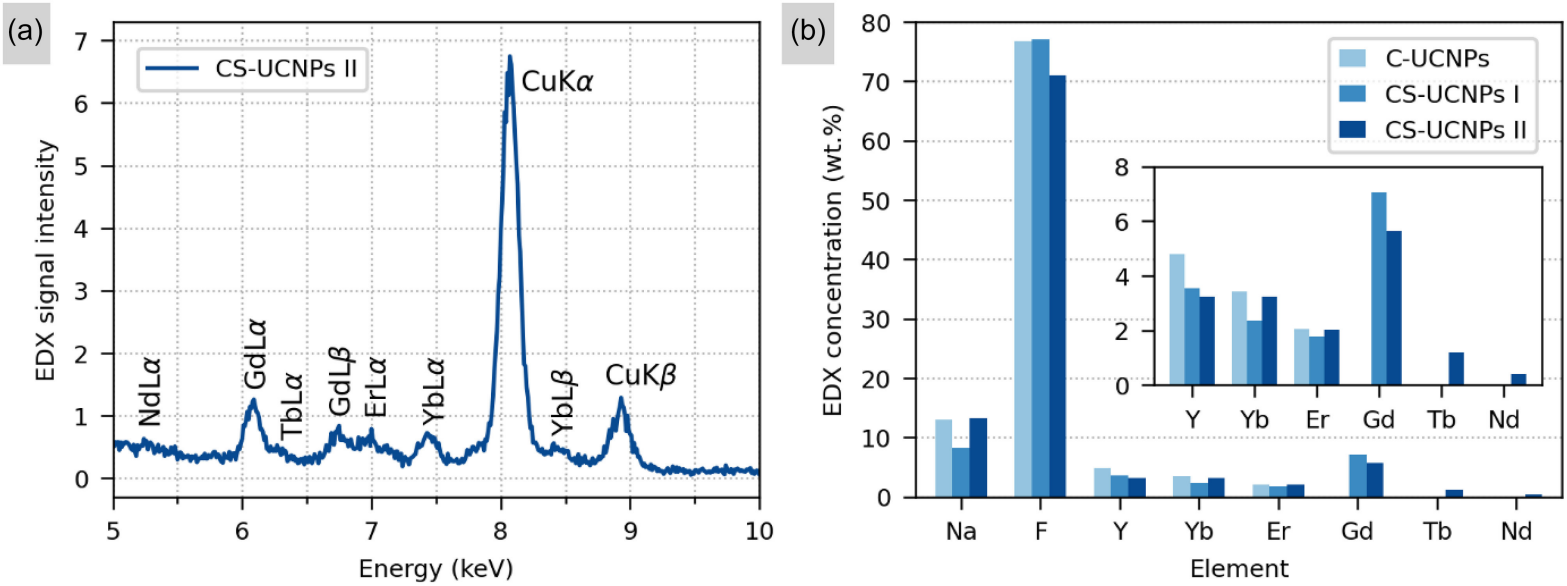
(a) TEM/EDX spectrum with peaks of rare‐earth elements and (b) elemental composition of C‐UCNPs (light blue), CS‐UCNPs I (medium blue), and CS‐UCNPs II nanoparticles (dark blue).

The elemental composition of the C‐ and CS‐UCNPs was verified by TEM/EDX analysis (Figure 3). All rare‐earth elements were detected (Figure 3a) and the expected composition of the nanoparticles was confirmed (Figure 3b). The concentration of iron was below the TEM/EDX detection limit, but the presence of Fe was proved by ICP‐MS technique (Table 2). Additionally, the ratios of rare‐earth ion concentrations in the C‐UCNPs, CS‐UCNPs I, and CS‐UCNPs II agreed with the stoichiometric ratios employed in the synthesis (Table 2). The measured elemental composition confirmed the incorporation of Yb, Er, Gd, Tb, Nd, and Fe ions into the corresponding core and core–shell systems. The increase in total Gd content after shell growth was consistent with NaGdF_4_ shell formation. The presence of Nd, Tb, and additional Yb in CS‐UCNPs II confirmed successful deposition of the NaGdF_4_:Nd,Yb,Tb shell. The differences in metal ion concentrations might be attributed to differences in particle mass and composition after shell growth, particle‐size/morphology distribution, and ICP‐MS matrix effects. This was consistent with the changes in ζ‐potential observed after shell formation (Table 1). For Fe ions in CS‐UCNPs I, small variations could have arisen from the analytical difficulty of Fe determination by ICP‐MS in complex fluoride nanoparticle digests because Fe monitoring is known to be affected by matrix‐related and polyatomic interferences [55].

**TABLE 2:** Metal ion concentration and relaxometry of nanoparticles at 37 °C and 1.5 T.

Particles	[Yb], mM	[Er], mM	[Fe], mM	[Gd], mM	[Tb], mM	[Nd], mM	∑, mM	*T*_1_, ms	*T*_2_, ms	*r*_1_, l/mmol⸱s	*r*_2_, l/mmol⸱s	*r*_2_/*r*_1_
C‐UCNPs	2.8 ± 0.05	1.8 ± 0.03	1.0^a^	—	—	—	5.6	3533 ± 58	179 ± 5	0.008	1.44	180
CS‐UCNPs I	0.97 ± 0.05	0.75 ± 0.02	0.32 ± 0.02	3.0 ± 0.1	—	—	5.0	338 ± 2	25 ± 1	0.61	8.5	13.9
CS‐UCNPs II	1.79 ± 0.10	0.93 ± 0.01	0.99 ± 0.13	3.1 ± 0.1	1.45 ± 0.06	0.42 ± 0.06	8.6	298 ± 19	42 ± 3	0.38	2.92	7.7
CS‐UCNP@PMVEMA	1.3^a^	0.9^a^	0.99 ± 0.13	3.1^a^	1.3^a^	0.3^a^	7.9	45 ± 1	12 ± 0.3	2.90	11.65	4.0

*Notes: T*_1,2_—relaxation times; *r*_1,2_—relaxivities.

a Concentrations determined from a single ICP‐MS measurement.

DLS measurements determined the hydrodynamic diameter of C‐UCNPs in water (*D*_h_ = 105 nm) and confirmed the low polydispersity of the particles (*PD* = 0.15; Table 1). The *D*_h_ was larger than *D*_n_ due to the presence of a solvated ionic layer around the particles causing a slight aggregation. The hydrodynamic diameters of the CS‐UCNPs I and II in water were 175 and 188 nm, respectively, with a moderate polydispersity (*PD* = 0.21 and 0.25; Table 1). The *D*_h_ of both CS‐UCNPs was larger than that of C‐UCNPs due to the presence of the NaGdF_4_ shell. The surface charge of C‐ and CS‐UCNPs I and II was positive (30, 23, and 31 mV, respectively) due to the presence of rare earth and iron cations on the particle surface.

### 3.2 Upconversion Luminescence of C‐ and CS‐UCNPs

**FIGURE 4:**
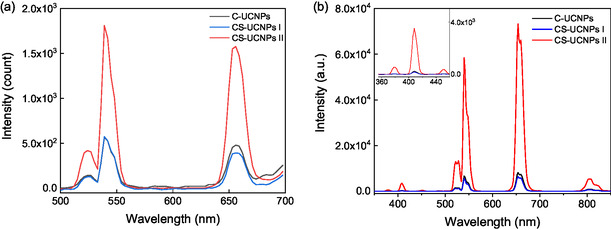
Upconversion photoluminescence spectra of C‐UCNPs, CS‐UCNPs I, and CS‐UCNPs II nanoparticles (1 mg/mL) after excitation at (a) 808 nm (3 W/cm^2^) and (b) 980 nm (2.11 W/cm^2^). The inset shows a scale‐up in the region <450 nm.

The upconversion luminescence emission of C‐ and CS‐UCNPs I and II was investigated under NIR excitation at 808 and 980 nm (Figure 4). Characteristic emission peaks originating from ^2^H_9/2_ → ^4^I_15/2_ (408 nm), ^2^H_11/2_ → ^4^I_15/2_ (522 nm), ^4^S_3/2_ → ^4^I_15/2_ (542 nm), ^4^F_9/2_ → ^4^I_15/2_ (654 nm), and 806 nm (^4^I_9/2_ → ^4^I_15/2_) transitions of Er^3+^ ions were observed in the spectra of all nanoparticles. The enhancement of the luminescence intensity in the red region originated from the presence of iron ions in the particles (Figure S2) [35]. Fe ions replacing lanthanide ions of the host crystal induced charge imbalance in the crystal lattices, reduced surface defects, and increased the intensity of red upconversion luminescence. The introduction of the inert NaGdF_4_ shell around the C‐UCNPs slightly affected the luminescence intensity under both 808 and 980 nm excitation, increasing the upconversion emission at 654 nm by 1.3‐fold compared to that of the C‐UCNPs. As expected, the largest luminescence intensity increase, making the upconversion emission in the red region dominant, was observed in the CS‐UCNPs II due to the presence of Tb^3+^, Nd^3+^, and Yb^3+^ ions in the NaGdF_4_ shell. Compared to the C‐UCNPs, the CS‐UCNPs II demonstrated 3 or 4 times higher upconversion intensity of green or red emission, respectively, after 808 nm excitation (Figure 4a). Under 980 nm excitation, the introduction of the NaGdF_4_:Yb,Tb,Nd shell increased the intensity of upconversion emission at 408, 522–544, 655, and 808 nm by 21×, 10×, 12×, and 8×, respectively (Figure 4b). The slight enhancement observed for CS‐UCNPs I compared to C‐UCNPs was attributed to the partial suppression of surface quenching by the NaGdF_4_ shell, whereas the significantly stronger emission of CS‐UCNPs II and the appearance of bands associated with Tb^3+^/Nd^3+^ indicated an additional contribution from the Nd^3+^‐, Yb^3+^‐, and Tb^3+^‐doped shell. Although the confirmed hexagonal β‐NaYF_4_/β‐NaGdF_4_ structure suggested that extensive cation migration should be limited, local interfacial redistribution of shell dopants, such as Tb^3+^ or Nd^3+^ near the core–shell boundary, cannot be fully excluded and may also influence the Yb^3+^–Er^3+^–Tb^3+^–Nd^3+^ energy‐transfer pathways. It was attributed to ^5^D_3_ → ^7^F_5_ (408 nm), ^5^D_4_ → ^7^F_5_ (544 nm), and ^5^D_4_ → ^7^F_2_ (650 nm) transitions of the characteristic Tb^3+^ emission and ^4^G_7/2_ → ^4^I_9/2_ (522 nm) and ^4^F_5/2_ → ^4^I_9/2_ (806 nm) transitions of Nd^3+^ emission involved in upconversion luminescence [14, 50]. The emission spectrum of CS‐UCNPs II nanoparticles exhibited new peaks at 380 and 450 nm after 980 nm excitation ascribed to ^5^D_3_ → ^7^F_6_ and ^5^D_4_ → ^7^F_4_ transitions of Tb^3+^ ions, respectively. Finally, the shells effectively isolated the core emitters from the external environment, significantly reducing surface‐related quenching effects and emission losses.

**FIGURE 5:**
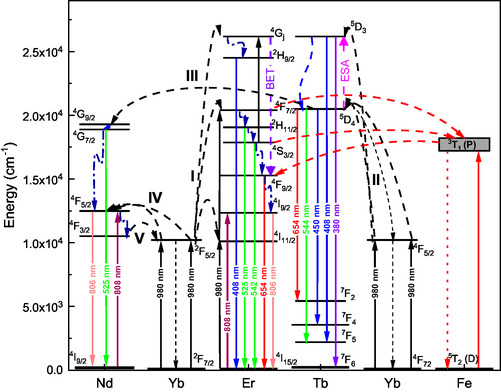
Possible energy transfer for CS‐UCNPs II nanoparticles excited at 808 and 980 nm. Only relevant energy levels with radiative (solid straight arrow) and nonradiative (blue dashed curved and red dotted arrow) transitions are depicted; the black (pathways *I*–*V*) and red dashed curved arrows represent possible energy transfer processes. BET   =  back‐energy transfer; ESA   =  excited state absorption. The NIST atomic spectrum database and Tanabe–Sugano energy diagram dependent on the crystal field strength were utilized to identify data and energy level spectral lines [56, 57].

The possible energy transfer processes in the CS‐UCNPs II excited at 808 and 980 nm can be interpreted using the energy transfer diagram based on the luminescence measurements (Figure 5). The most efficient upconversion mechanism for C‐ and CS‐UCNPs was considered to be the energy transfer upconversion (ETU), involving multiple competing transitions between several energy levels of Yb^3+^–Er^3+^ in the NaYF_4_ host [58]. The introduction of transition metal ions into NaYF_4_:Yb,Er crystal resulted in a novel process of energy transfer between Er^3+^ and Fe^2+^ ions [59]. The energy levels of Fe ions in the fluoride host of C‐UCNPs depended on the strength of the crystal field, decreasing radiative transition rate of Er^3+^ from ^4^S_3/2_ and ^2^H_11/2_ levels to ^4^I_15/2_ ground state (Figure 5). At the same time, the population density of ^3^T_1_ level of Fe ions increased due to resonant energy transfer. Fe ions inducing intermediate energy states facilitated energy transfer between the Yb sensitizer and the Er activator, thereby increasing the probability of forbidden *f*–*f* transitions in Er^3+^ and Yb^3+^ ions and providing additional efficient energy transfer pathways (Figure 5; red dashed line). It may also originate from the sensitization via the Yb^3+^–Fe^2+^ dimer complex, which is quite similar to the recent reports on the enhancement of luminescence efficiency in the Yb^3+^‐transition metal ion dimer system [60, 61].

At 980‐nm excitation of CS‐UCNPs II, the incident photons were absorbed by Yb^3+^ ions at ^2^F_7/2_ ground state, promoting them to ^2^F_5/2_ excited state (Figure 5; solid black lines). The part of energy relaxed to the ^2^F_7/2_ ground state of Yb^3+^ via nonradiative or radiative decay. Subsequently, the energy from ^2^F_5/2_ (Yb^3+^) was resonantly transferred to the neighboring Er^3+^ ion through ETU process (pathway I) populating its ^4^I_11/2_ state as a result of the matching of excited energy states. This level of Er^3+^ ion could be also populated by direct excitation of Er^3+^ ion from its ^4^I_15/2_ state by ground state absorption process. An additional energy transfer occurred from another Yb^3+^ to the Er^3+^, resulting in further excitation of Er^3+^ of the higher ^4^F_7/2_ level. The higher energy states ^4^G_j_ (J = 7/2, 9/2, and 11/2) of Er^3+^ were excited through multiphonon relaxation and Yb‐to‐Er energy transfer. The red emitting level (^4^F_9/2_ of Er^3+^) was also generated by the back‐energy transfer (BET) process between ^4^G_j_ (Er^3+^) and a ground state of Yb^3+^ and Er^3+^ ions. Then, Er^3+^ ions decayed nonradiatively to the ^2^H_9/2_, ^2^H_11/2_, ^4^S_3/2_, ^4^F_9/2_, and ^4^I_9/2_ luminescent states emitting at 408, 525, 542, 654, and 806 nm, respectively. Additionally, a pair of excited Yb^3+^ ions transferred their energy to the nearby upper ^5^D_4_ excited state of Tb^3+^ ions via cooperative energy transfer (pathway II) and then radiatively relaxed to lower ^7^F levels by emitting visible photons at 450 nm (^5^D_4_ → ^7^F_4_), 544 nm (^5^D_4_ → ^7^F_5_) and 654 nm (^5^D_4_ → ^7^F_2_) [62]. A part of electrons could be pumped from ^5^D_4_ to ^5^D_3_ level of Tb^3+^ by the ETU from Yb^3+^ or by the excited state absorption, producing ^5^D_3_ → ^7^F_6_ and ^5^D_3_ → ^7^F_5_ emissions at 380 and 408 nm, respectively. Additionally, a nonradiative energy could be transferred from the ^5^D_4_ level of Tb^3+^ to the ^4^G_9/2_ level of Nd^3+^ ions via BET (pathway III). Then, the ^4^G_9/2_ state was relaxed nonradiatively to lower ^4^G_7/2_ and ^4^F_5/2_ states through multiphonon relaxation. The ^4^F_5/2_ level of Nd^3+^ could also be occupied by the cooperative or phonon‐assisted energy transfer from two adjacent Yb^3+^ ions (pathway IV). The radiative depopulation of these levels to the ground state led to upconversion emissions at 525 and 806 nm cor‐responding to ^4^G_7/2_  →  ^4^I_9/2_ and ^4^F_5/2_  →  ^4^I_9/2_ transitions of Nd^3+^ ions, respectively.

At 808‐nm excitation of CS‐UCNPs II, the Nd^3+^ and Er^3+^ ions were first excited from their ground states to the higher ^4^F_5/2_ and ^4^I_9/2_ states, respectively [63]. The excited Nd^3+^ ions then quickly relaxed to the ^4^F_3/2_ level through phonon‐assisted relaxation. A part of this energy was then resonantly transferred to ^2^F_5/2_ energy level of Yb^3+^ (Figure 5; pathway V), acting as a bridge between Nd^3+^ and Er^3+^ ions, while the remaining energy decayed radiatively to the ^4^I_9/2_ ground state of Nd^3+^. Afterward, it resonantly transferred the energy to the neighboring Er^3+^ and Tb^3+^ ions, exhibiting the red and green emissions in the same manner as described previously for 980‐nm excitation.

### 3.3 Relaxometry of C‐ and CS‐UCNPs

**FIGURE 6:**
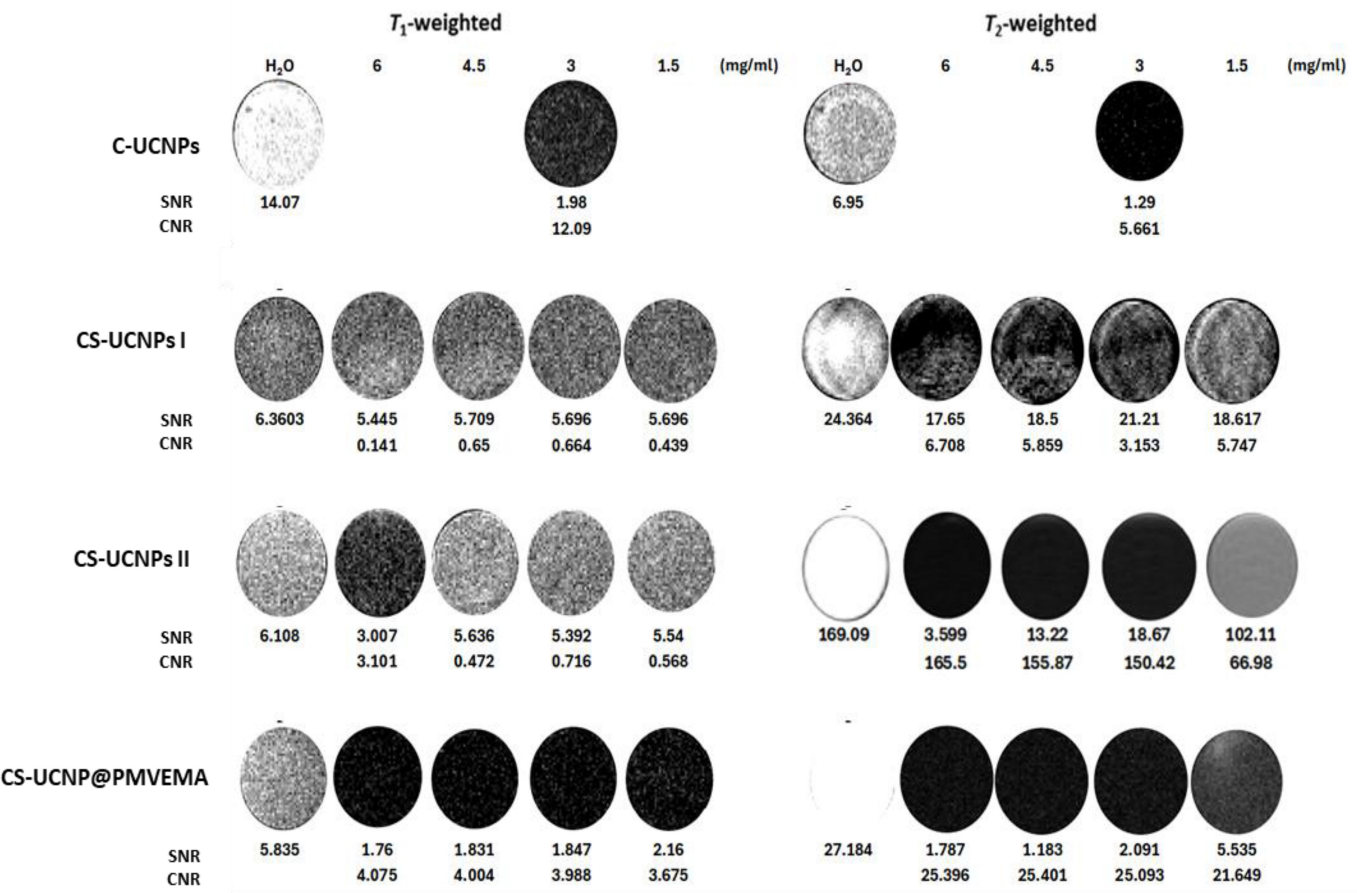
*T*_1_‐ and *T*_2_‐weighted MR images of phantoms containing C‐UCNPs, CS‐UCNPs I, CS‐UCNPs II, and CS‐UCNP@PMVEMA nanoparticles (1.5–6 mg/mL) on a Bruker scanner with surface coil magnetic field *B*_0_ = 7 T. C‐UCNPs were imaged in gelatin (3 mg/mL) due to their instability in water. The corresponding SNR and CNR were calculated from MR images with one acquisition. The water phantom was used as a reference.

Key parameters defining the MRI contrast of C‐ and CS‐UCNPs included the longitudinal (*r*_1_) and transverse (*r*_2_) relaxivities, as well as the *r*_2_/*r*_1_ ratios, derived from the relaxation times plotted against lanthanide and iron ion concentration (Table 2; Figure S3). The relaxivities of the aqueous particle dispersions showed that all nanoparticles exhibited *T*_2_‐weighted contrast properties. The *r*_1_ relaxivity of C‐UCNPs was low (∼0.01 l/mmol·s^‒1^) as Y^3+^, Yb^3+^, and Er^3+^ ions had a negligible effect on *T*_1_ relaxation times. The resulting *r*_2_/*r*_1_ ratio remained quite high and close to 180. The *T*_1_ relaxation depended mainly on the Gd^3+^ content in the nanoparticles, while the whole crystal structure of the NaYF_4_ core and Fe ions contributed to the *T*_2_ relaxation. Compared to the relaxivities of the C‐UCNPs, the presence of undoped and Nd‐, Yb‐, and Tb‐doped NaGdF_4_ shells increased the *r*_2_ relaxivities to 8.5 and 2.92 l/mmol·s^‒1^, respectively. The CS‐UCNPs I and II containing Gd in the shell exhibited ∼75‐ and ∼50‐fold higher *r*_1_ relaxivity, respectively, and had a low *r*_2_ */r*_1_ ratio (Table 2). The suitability of C‐ and CS‐UCNPs for MRI was also evaluated at human body temperature (37 °C) by acquiring *T*_1_‐ and *T*_2_‐weighted images of aqueous particle dispersions with concentrations ranging from 1.5 to 6 mg/mL (Figure 6). The imaging results were compared to those of the relaxometry results. The *T*_1_‐weighted MRI phantoms showed only a small change in the signal intensity due to the predominance of the *T*_2_ effect associated with high metal concentrations. All tested particle concentrations demonstrated marked *T*_2_ contrast, appearing distinctly hypointense in *T*_2_‐weighted MR imaging of the phantom samples. Based on the good performance of luminescence and phantom imaging of monodisperse CS‐UCNPs II, they were selected for their modification by a hydrophilic PMVEMA coating and in vitro and in vivo investigation.

### 3.4 Coating of CS‐UCNPs II With PMVEMA

Since the synthesized CS‐UCNPs II were hydrophobic, it was crucial to modify them with a biocompatible PMVEMA layer to make them water‐dispersible and colloidally stable without aggregation or precipitation [36]. The successful modification of the particles was documented by TGA, ATR‐FTIR spectroscopy, and DLS (Table 1; Figure S4). The resulting CS‐UCNP@PMVEMA nanoparticles contained 4.7 wt.% PMVEMA on the surface (Figure S4a). Small weight losses of the neat and polymer‐coated CS‐UCNPs II nanoparticles at heating from RT to 200 °C were ascribed to the evaporation of residual water. The ATR‐FTIR spectrum of the CS‐UCNPs II particles showed very weak peaks at 2925, 2855, 1636, and 1565 cm^–1^ attributed to the asymmetric ν_as_(CH_2_), symmetric ν_s_(CH_3_) and ν(C═C), and asymmetric ν_as_(COO^–^) stretching vibrations of residual oleic acid remaining after washing, respectively (Figure S4b). After the modification of CS‐UCNPs II with PMVEMA, the spectrum confirmed the presence of the polymer on the nanoparticle surface, where the characteristic asymmetric (1573 cm^−1^) and symmetric (1435 cm^−1^) stretching ν(C═O) vibrations of the COOH groups were found. The shoulder at ~1700 cm^−1^ and new band at 1081 cm^−1^ were attributed to the ν(C═O) and ν(C─O) stretching vibrations. The change in the ζ‐potential, which became negative, was associated with the presence of carboxyl groups of PMVEMA on the particle surface (ζ‐potential = −28 mV; Table 1). After coating, the hydrodynamic diameter of CS‐UCNP@PMVEMA particles in water decreased to 149 nm with low polydispersity (*PD* = 0.16), which was attributed to the efficient electrostatic stabilization provided by the hydrated PMVEMA layer. The *D*_h_ was smaller than that of the uncoated particles due to a good stabilizing efficiency of PMVEMA preventing aggregation. The colloidal stability of CS‐UCNP@PMVEMA particles was further investigated in 0.01 M PBS buffer, 0.9% NaCl (physiological solution), and FBS‐supplemented CMRL medium used in animal experiments. The *D*_h_ of the particles incubated in the CMRL medium for 1 h was 133 nm (*PD* = 0.21) and increased after 72 h to 167 nm (*PD* = 0.28), probably due to partial rearrangement of the polymer layer and initial adsorption of proteins and other biomolecules on the particle surface. The hydrodynamic diameters of the particles in PBS and saline were 233 nm (*PD* = 0.19) and 105 nm (*PD *= 0.13) and remained fairly constant for 72 h of storage without any sign of sedimentation. Salts and phosphate ions can screen the negative charge of carboxyl groups and change the conformation of the hydrated polymer layer, which demonstrated superior colloidal stability of CS‐UCNP@PMVEMA particles. The PMVEMA coating slightly decreased the upconversion emission intensity of CS‐UCNPs II particles under NIR excitation at 808 and 980 nm due to increased nonradiative relaxation at the nanoparticle surface caused by interaction with water molecules (Figure S5).

### 3.5 MR Relaxometry and Imaging of Phantoms Containing CS‐UCNP@PMVEMA Nanoparticles

After the modification of CS‐UCNPs II with PMVEMA, the particles exhibited high *r*_1_ and *r*_2_ relaxivities, with a clearly detectable *T*_2_‐weighted contrast component (Table 2; Figure S3). The *T*_1_ contrast of CS‐UCNP@PMVEMA nanoparticles was masked by the dominant strong *T*_2_ effect at all tested concentrations under magnetic field *B*_0_ = 7 T. Therefore, under the applied imaging conditions, the observed MRI contrast reflected susceptibility‐driven *T*_2_/*T*_2_* effects. As a result, the *T*_2_ contrast remained clearly distinguishable even at the lowest particle concentration (1.5 mg/mL; Figure 6). Their longitudinal (*r*_1_ = 2.9 l/mmol·s^‒1^) and transverse (*r*_2_ = 11.7 l/mmol·s^‒1^) relaxivities were nearly 8 and 4 times higher, respectively, than those of uncoated CS‐UCNPs II with low *r*_2_/*r*_1_ ratio amounting to 4.0 (Table 2). The remarkably good dispersibility and colloidal stability of CS‐UCNP@PMVEMA particles in aqueous media were key to their high *r*_1_ and *r*_2_ relaxivities (Table 2). The coating made the surface of the nanoparticles hydrophilic and accessible to water protons, enabling water molecules to interact with the paramagnetic lanthanide and iron dopants on the particle surface efficiently. The relaxation was comparable to that of PEG‐neridronate‐coated NaYF_4_:Gd, Yb,Tm@NaGdF_4_ CS‐UCNPs (~26 nm) used as a bimodal luminescent/MRI agent [64]. After coating with a hydrophilic PEG‐bisphosphonate, both *r*_1_ and *r*_2_ increased significantly (while still maintaining *r*_2_ > *r*_1_), indicating the crucial role of surface functionalization in enabling dual contrast. In summary, MRI phantoms of aqueous CS‐UCNP@PMVEMA particle dispersions showed 15‐fold higher MR signal compared to those obtained for water phantoms. SNR and CNR calculated from MR images indicated that the CS‐UCNP@PMVEMA nanoparticles could provide sufficient MR contrast, enabling the detection of labeled Langerhans islets.

**FIGURE 7:**
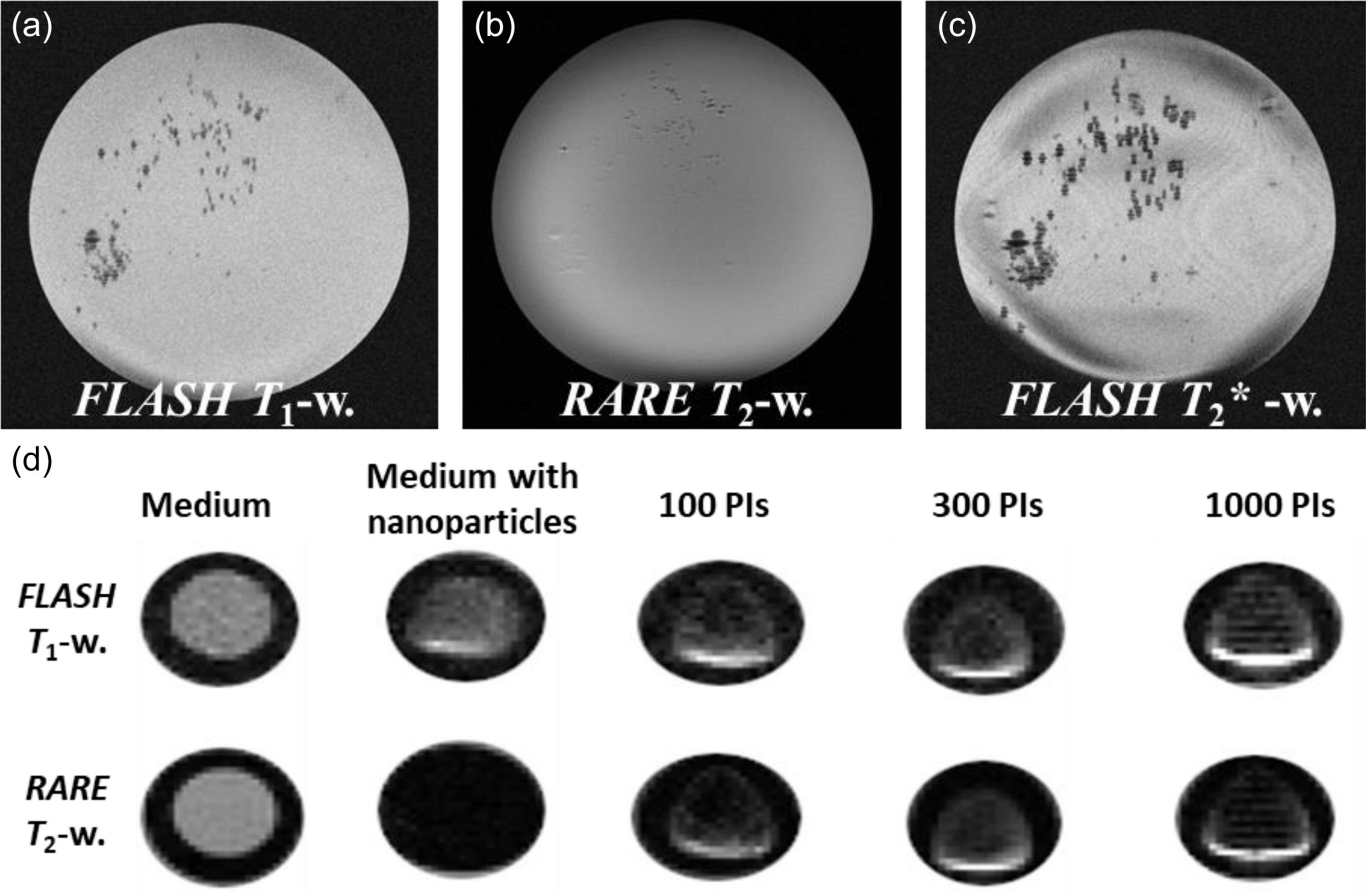
In vitro (a) *T*_1_‐, (b) *T*_2_‐, and (c) *T*_2_*‐weighted MRI of CS‐UCNP@PMVEMA‐labeled pancreatic Langerhans islets (PIs). (d) Concentration‐dependent FLASH and RARE *T*_1_ ‐ and *T*_2_‐weighted MRI contrast of CS‐UCNP@PMVEMA‐labeled Langerhans islets with the CMRL medium phantom as a reference.

To evaluate MRI performance in vitro, Langerhans islets were labeled with CS‐UCNP@PMVEMA nanoparticles embedded in gelatin phantoms and scanned at 7 T (Figure 7). The labeled islets demonstrated substantial alterations in both *T*_1_ and *T*_2_ relaxation times, resulting in pronounced contrast on RARE and FLASH *T*_1_‐, *T*_2_‐, and *T*_2_*‐weighted imaging sequences (Figure 7a–c). Both *T*_1_‐ and *T*_2_‐weighted images showed predominantly *T*_2_‐weighted hypointense signal, indicating that the *T*_1_ shortening‐induced hyperintensity was masked by the dominant *T*_2_ contribution. This suggested that low nanoparticle concentrations would be more suitable for achieving a primarily *T*_1_‐weighted positive contrast, which was consistent with the phantom relaxometry experiments. Unlabeled islets embedded within the same phantoms served as controls, enabling direct comparison and highlighting the clear impact of nanoparticle labeling. In vitro evaluation of concentration‐dependent MRI contrast of the CS‐UCNP@PMVEMA particle‐labeled Langerhans islets proved the significant *T*_2_ shortening effect of the particles in a stable reference environment (Figure 7d). The *T*_1_‐weighted MRI contrast was strongest in the FLASH sequences at the lowest number of labeled pancreatic islets, which was consistent with *T*_1_ shortening. These results confirmed that CS‐UCNP@PMVEMA nanoparticles provided strong and consistent signal modulation, which supports their suitability for high‐contrast detection of islet grafts. Overall, the combined relaxometry and imaging results demonstrated that CS‐UCNP@PMVEMA nanoparticles provided strong MRI signal modulation, with measurable *T*_1_‐shortening properties but predominantly *T*_2_/*T*_2_*‐weighted contrast at both clinical (1.5 T) and high‐field strengths (7 T), making them ideal for noninvasive monitoring of transplanted islets and other cell therapies.

### 3.6 Toxicity and Optical Imaging of CS‐UCNP@PMVEMA‐Labeled Langerhans Islets

**FIGURE 8:**
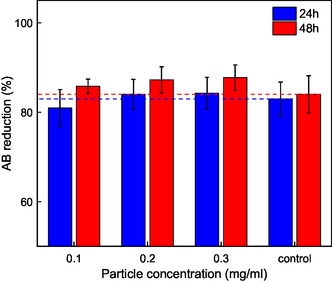
Concentration‐dependent viability of murine mammary carcinoma 4T1 cells incubated with CS‐UCNP@PMVEMA nanoparticles for 24 and 48 h determined by Alamar Blue (AB) reduction method. The AB reduction (%) indicates the proportion of resazurin converted to resorufin by viable cells. It was compared to the percentage reduction in the positive control (cells not exposed to particles; dashed line) to assess the cytotoxicity of the particles. Data are presented as mean ± SD from three independent experiments, each performed in technical triplicate; error bars represent SD.

To evaluate the potential of CS‐UCNP@PMVEMA nanoparticles for diagnostics in diabetology, it was essential to examine their toxicity. In vitro cytotoxicity of the nanoparticles was tested using nondestructive Alamar Blue reduction assay after 24 and 48 h of incubation with murine mammary carcinoma 4T1 cells, as a standard, robust, syngeneic mouse model (Figure 8). This cell‐viability assay quantifies metabolic activity via the conversion of nonfluorescent resazurin to fluorescent resorufin in the cellular reducing environment, providing a robust readout for drug screening and nanoparticle safety at the cellular level [65]. Exposure of murine mammary carcinoma 4T1 cells to CS‐UCNP@PMVEMA nanoparticles (0.1–0.3 mg/mL) did not alter their cell viability after 24 and 48 h. Compared to the nontreated cells, no significant differences were found after 48 h of incubation, even at a maximum concentration of 0.3 mg/mL, documenting high cell viability. Compared with NaGdF_4_:Yb,Tb,Nd nanoparticles coated with poly(4‐styrenesulfonic acid‐*co*‐maleic anhydride) used for pancreatic islet and β‐cell imaging, which induced cytotoxicity at 0.1 mg/mL after 24 h, PMVEMA coating exhibited clearly improved biocompatibility [14]. The cytotoxic effect of CS‐UCNP@PMVEMA particles was similar to previously developed perfluorocarbon and iron oxide nanoparticles for pancreatic β‐cell and islet imaging [66, 68].

In pancreatic islet transplantation, maintaining high islet viability is crucial for successful healing and long‐term function. Langerhans islets labeled with CS‐UCNP@PMVEMA nanoparticles in vitro showed a decrease in viability to 88% ± 3% after 20 h compared to 96 ± 1% for control unlabeled islets (*p* < 0.01). Viability above 70% is generally considered acceptable for islet transplantation, as it still ensures reliable evaluation of islet quality [69]. Moreover, recent advances in cryopreservation techniques have achieved postthaw viabilities of ∼87% in human islets, confirming that such a level was compatible with functional islet preparations [70]. It should be noted that the higher nanoparticle concentrations used for MRI phantom measurements were acellular and therefore did not represent direct cellular exposure, whereas the biologically relevant islet‐labeling protocol involved incubation of intact islets with CS‐UCNP@PMVEMA nanoparticles followed by washing before imaging or transplantation.

**FIGURE 9:**
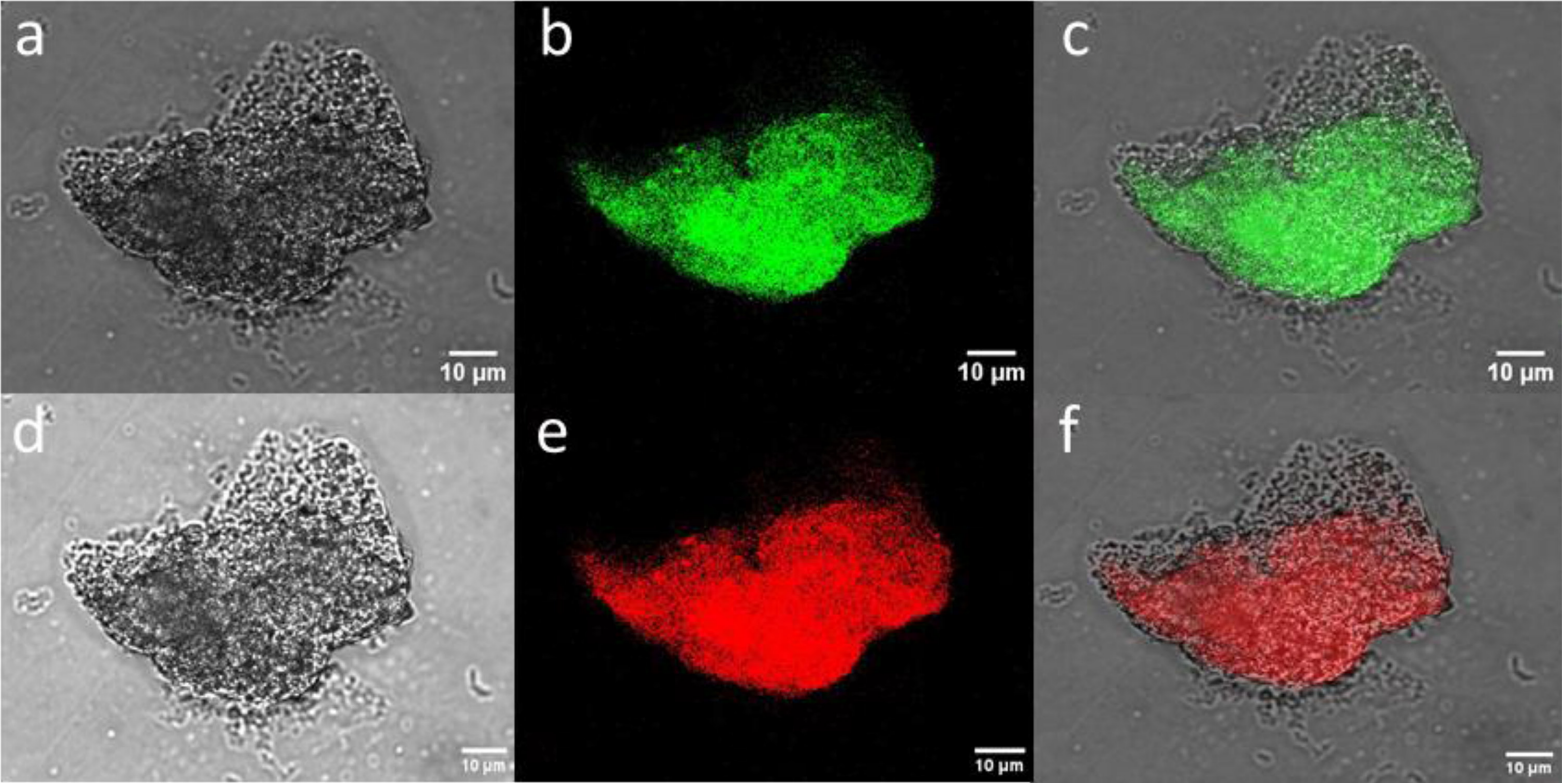
In vitro confocal micrographs of CS‐UCNP@PMVEMA‐labeled Langerhans islet: (a,d) bright‐field images at 405 nm, (b,e) upconversion luminescence under 980 nm (b) and 808 nm (e), and (c,f) merged overlays.

In vitro labeling of living Langerhans islets incubated with CS‐UCNP@PMVEMA nanoparticles for 20 h was demonstrated by confocal imaging at both 980 and 808 nm excitation (Figure 9). The Langerhans islets were easily visualized in bright‐field mode by transmitted light and due to the upconversion luminescence of the particles. The overlay of micrographs demonstrated the distribution of particles inside the Langerhans islets, thus confirming the binding of nanoparticles to isolated pancreatic islet cells. Moreover, confocal imaging at 808 (Figure 9e,f) and 980 nm (Figure 9b,c) excitations further confirmed that CS‐UCNP@PMVEMA nanoparticles maintained their dual excitability in the NIR range after the islet labeling, thus providing an additional excitation channel for upconversion imaging in the transparent biological window. This may enable precise visualization of the transplanted pancreatic islets, facilitating tracking after transplantation and supporting the noninvasive assessment of graft localization and integrity.

### 3.7 In Vivo MRI of CS‐UCNP@PMVEMA‐Labeled Langerhans Islets

**FIGURE 10:**
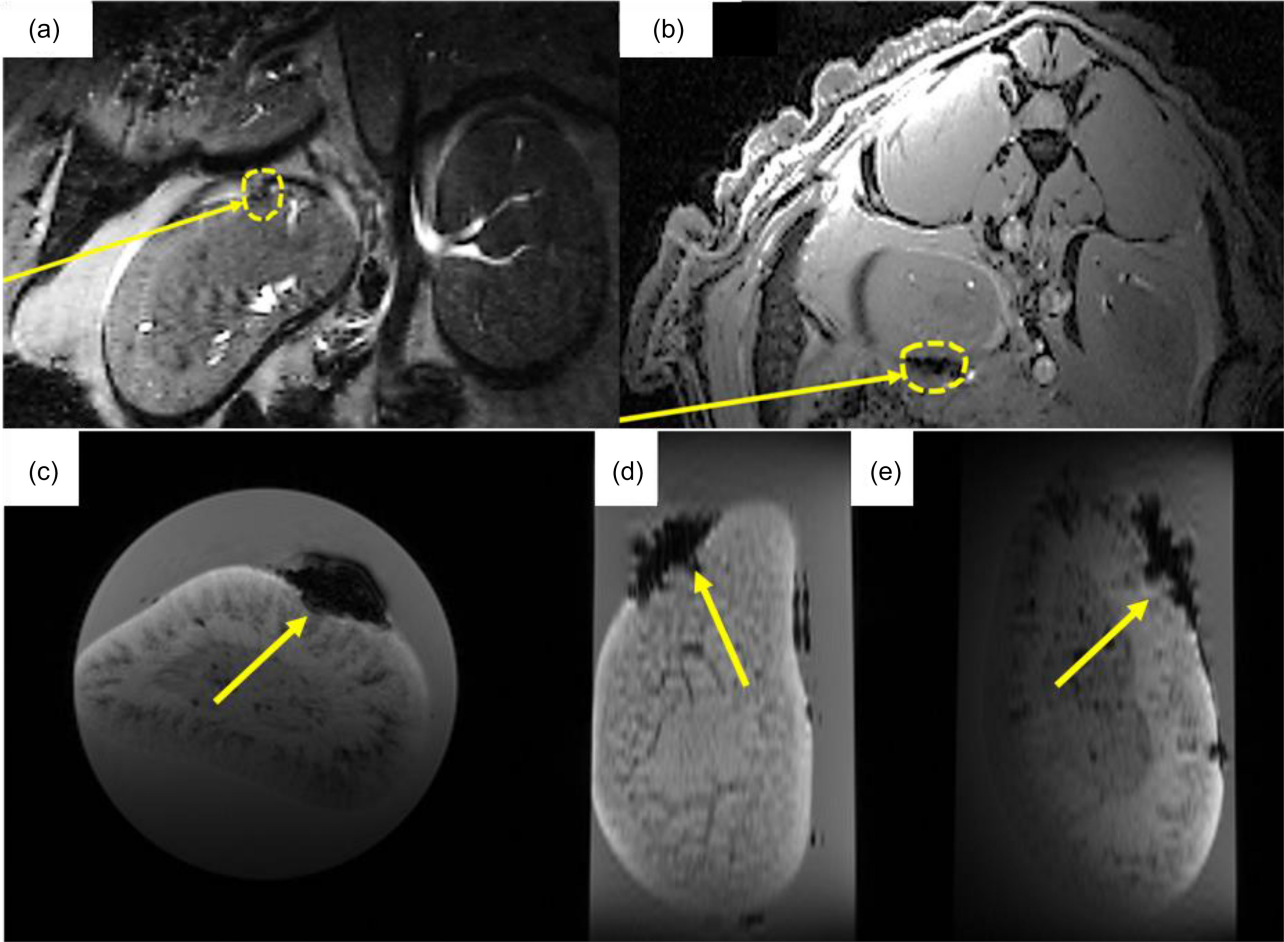
In vivo (a,b) *T*_2_*‐ and (c–e) *T*_1_‐weighted 3D FLASH MRI of CS‐UCNP@PMVEMA‐labeled rat pancreatic Langerhans islets transplanted under the kidney capsule (a,b) before and (c–e) after explantation: (a,e) coronal, (b,c) axial, and (d) sagittal views. Yellow arrows show the transplanted nanoparticle‐labeled pancreatic islets. CNRs calculated relative to the kidney cortex were 26.2 (a), 53.3 (b), and 256.3 (c–e; orthogonal views from the same 3D scan).

*T*_1_‐ and *T*_2_*‐weighted MRI were employed to evaluate the in vivo visualization performance of CS‐UCNP@PMVEMA‐labeled Langerhans islets transplanted under the renal capsule of a rat (Figure 10). To ensure maximal labeling efficiency, Langerhans islets were labeled using the highest tested nanoparticle concentration (2.3 mg/mL). Gradient echo *T*_2_*‐weighted sequences, with their increased sensitivity to susceptibility effects and short repetition time, were specifically chosen for post‐transplantation to reduce motion artifacts caused by respiration and peristalsis in an animal model (Figure 10a,b). In vivo MRI distinctly demonstrated the particle‐labeled islets as hypointense regions clearly distinguishable from surrounding tissues, reflecting strong signal contrast. Importantly, control animals without transplanted labeled islets showed no comparable hypointense signals, supporting the attribution of the observed signal loss to the transplanted labeled islets (data not shown). Despite employing 500 labeled islets, which is significantly lower compared to clinical islet transplantation protocols used in the treatment of type 1 diabetes, effective visualization at a magnetic field strength of 7 T emphasized the high sensitivity and efficiency of this imaging approach. Furthermore, MRI was extended to evaluate particle‐labeled Langerhans islets in an in vivo context after transplantation under the renal capsule of a rat. A strong *T*_2_*‐weighted effect dominated both *T*_1_‐ and *T*_2_*‐weighted images, leading to a predominantly negative (hypointense) appearance, thereby substantiating prior in vitro assessments. Accordingly, under the used in vivo conditions, the MRI of the labeled islets was governed mainly by susceptibility‐driven *T*_2_/*T*_2_* effects, while the *T*_1_ contribution was largely masked. Although negative contrast may be less specific than positive enhancement on *T*_1_‐weighted images, since areas of signal loss may also originate from hemorrhage, vessels, calcifications, air/tissue interfaces, or local magnetic field inhomogeneities, susceptibility‐based *T*_2_/*T*_2_*‐weighted imaging remains highly sensitive for tracking prelabeled islet grafts in defined anatomical sites. This strategy has been widely used for experimental and clinical visualization of labeled pancreatic islets, supporting the relevance of the predominantly hypointense contrast observed in the present study.

*T*_1_‐weighted MRI postnephrectomy further corroborated the in vivo observations and offered detailed anatomical insights beyond the resolution achievable through whole‐animal imaging alone (Figure 10c–e). The transplanted islets were detected with a very high CNR of over 25, reflecting the high efficiency of the developed particles. This highlighted the strong potential of CS‐UCNP@PMVEMA nanoparticles as a safe and effective dual *T*_1_‐ and *T*_2_*‐weighted MRI contrast agent with predominant *T*_2_/*T*_2_* effects for in vivo monitoring of islet transplantation.

Conventional lanthanide‐based contrast agents primarily demonstrate either *T*_1_ or *T*_2_ contrast enhancement, with limited examples of dual‐mode capability. Traditional gadolinium diethylenetriamine pentaacetic acid exhibited strong *T*_1_ contrast but was associated with nephrogenic systemic fibrosis and cannot be used, repetitively, in compromised renal function [71]. Well‐established iron oxide‐based *T*_2_ contrast agents typically caused pronounced susceptibility effects due to the negative contrast of the particles against the background of body tissues, limiting anatomical clarity and misleading clinical diagnosis [72]. The deep anatomical location of pancreas, surrounded by complex structures such as the intestines, bile ducts, and blood vessels, can further introduce artifacts and signal noise in *T*_2_*‐weighted MRI images, compromising image quality and quantification. Moreover, the limited usage of iron oxide nanoparticles was due to their high transverse to longitudinal relaxivity ratio [73].

Multimodal probes have been explored to overcome these limitations in pancreatic islet transplantation. The modification of superparamagnetic iron oxide nanoparticles with a NIR fluorescent dye provided MR detectability and NIR fluorescence tracking of labeled human or rodent islets in subrenal capsule and intraportal models [74]. Poly(lactic‐*co*‐glycolic acid)‐based nanoparticles embedding perfluorocarbon and fluorophores combined specific quantitative ^19^F MRI with sensitive optical imaging, providing additional information for determining the distribution and survival of islet grafts [75]. Gd‐doped Fe_3_O_4_ nanoparticles coated with bovine serum albumin demonstrated high specificity in liver disease models, providing dual‐mode *T*_1_ and *T*_2_ MRI contrast with the low relaxivity ratio (*r*_2_/*r*_1_ = 10.7), which improved diagnostic speed and precision [76].

In comparison with our previous poly(4‐styrenesulfonic acid‐*co*‐maleic anhydride)‐coated NaGdF_4_:Yb,Tb,Nd nanoparticles for multimodal imaging of pancreatic Langerhans islets and β‐cells, uniform dumbbell‐shaped CS‐UCNPs provided brighter light emission in the red region at both 980 and 808 nm excitation, making the particles suitable for deep tissue imaging [14]. However, for future in vivo optical imaging, 808 nm excitation is expected to be preferable because it minimizes water‐mediated heating compared with 980 nm irradiation. PMVEMA coating improved colloidal stability in physiological media and maintained islet viability after labeling. The large number of carboxyl groups of PMVEMA also makes it possible to immobilize biomolecules for theranostic applications in the future. In addition, adopting the rat renal capsule model not only increased graft capacity but also minimized partial‐volume artifacts across surrounding tissues, thereby enhancing MRI quantification. Here, the developed multimodal CS‐UCNP@PMVEMA nanoparticles combined upconversion luminescence for high‐resolution islet‐level verification with MRI detection suitable for longitudinal in vivo tracking while directly addressing sensitivity issues in small subcapsular grafts. Future work should assess in vivo optical imaging and MRI of labeled islets at clinically relevant sites (e.g., intraportal) in order to fully realize the potential applications of CS‐UCNP@PMVEMA nanoparticles and to validate translation. Similarly, systematic histopathological analysis of major organs and long‐term biosafety evaluation will be required in further studies to assess the translational potential of the nanoparticles, especially for systemic administration or repeated in vivo applications.

## 4 Conclusions

Novel dumbbell‐shaped NaYF_4_:Yb,Er,Fe@NaGdF_4_:Nd,Yb,Tb CS‐UCNPs with a uniform size, regular morphology, and good crystallinity required for bioapplications were successfully synthesized. The customizable incorporation of lanthanide ions such as Gd^3+^, Er^3+^, Tb^3+^, Nd^3+^, and Yb^3+^ together with Fe dopants enabled precise tuning of both relaxivity and luminescence properties. The introduction of PMVEMA coating notably enhanced both the biocompatibility and colloidal stability of the nanoparticles, which is a key factor for their successful clinical translation. Importantly, the CS‐UCNP@PMVEMA nanoparticles possessed combined strong MRI contrast with NIR‐mediated upconversion luminescence, thus paving the way for multimodal imaging strategies. Phantom imaging of nanoparticle‐labeled Langerhans islets confirmed the strong and consistent modulation of *T*_1_‐, *T*_2_‐, and *T*_2_*‐weighted MR signals in vitro and in vivo, demonstrating their capability for effective visualization of islet grafts. However, susceptibility‐driven *T*_2_/*T*_2_ * effects dominated over *T*_1_ shortening, resulting predominantly in hypointense negative contrast. In vivo experiments in a rat model further validated these observations, demonstrating clear and reproducible detection of the transplanted particle‐labeled Langerhans islets, even at significantly reduced graft volumes. Standard gradient echo MRI distinctly revealed labeled islets as hypointense regions, with in vitro imaging of explanted kidneys corroborating signal retention and stability. Thus, CS‐UCNP@PMVEMA nanoparticles offer substantial promise as a noninvasive MRI contrast agent for pancreatic islet transplantation, potentially extending their utility to other cell‐based therapeutic applications. Collectively, these findings underscore their considerable clinical potential for early diagnosis, prognostic evaluation, and monitoring of therapeutic efficacy and recurrence in clinical settings.

## Author Contributions

The manuscript was written through contributions of all authors. All authors have given approval to the final version of the manuscript.

## Funding

This work was supported by the Grantová Agentura České Republiky (24‐10125S).

## Conflicts of Interest

The authors declare no conflicts of interest.

## Supporting information

Supplementary Material

## Data Availability

The data that support the findings of this study are available from the corresponding author upon reasonable request.
