# Supplementary material for: Upconverting Nanoparticles for Bimodal Luminescence and Magnetic Resonance Imaging of Langerhans Islets
Source: Small Sci. 2026 Jul 16;6(7):e70344. doi: 10.1002/smsc.70344 (PMC13387338; doi:10.1002/smsc.70344)
Supplement: Supplementary file 1 — Supplementary Material [file SMSC-6-e70344-s001.pdf]

Supporting Information

Upconverting nanoparticles for bimodal luminescence and magnetic resonance imaging of Langerhans islets

Oleksandr Shapoval, Daniel Jirák, Zuzana Berková*, Miroslav Šlouf, Olga Kočková, Hana Engstová, Aminadav Halili, David Červený, Petr Ježek, Daniel Horák**

O. Shapoval, M Šlouf, O. Kočková, D.Horák
Institute of Macromolecular Chemistry of the Czech Academy of Sciences, Heyrovského nám. 2, 162 00 Prague 6, Czech Republic
E-mail: shapoval@imc.cas.cz, horak@imc.cas.cz

D. Jirák, Z. Berková, A. Halili, D. Červený,
Institute for Clinical and Experimental Medicine, Vídeňská 1958/9, 140 21 Prague 4, Czech Republic
E-mail: zube@ikem.cz

D. Jirák, D. Červený
Institute of Biophysics and Informatics, First Faculty of Medicine, Charles University, 121 08 Prague 1, Czech Republic

D. Jirák,
Faculty of Health Studies, Technical University of Liberec, 461 17 Liberec, Czech Republic

H. Engstová, P. Ježek
Institute of Physiology of the Czech Academy of Sciences, Vídeňská 1083, 142 20 Prague 4, Czech Republic

Table S1. Parameters of *in vitro* T_1- and T_2-weighted magnetic resonance imaging of CS-UCNP@PMVEMA nanoparticles in water phantoms and Langerhans islets.

	T_1				T_2			
	TR (ms)	TE (ms)	Averages	Scan time (s)	TR (ms)	TE (ms)	Averages	Scan time (s)
RARE	1250	15	1	600	2500	40	1	600
FLASH	250	7	5	600	1250	30	1	600

TR – repetition time, TE – echo time. RARE - rapid acquisition with relaxation enhancement, FLASH - gradient echo fast low angle shot.

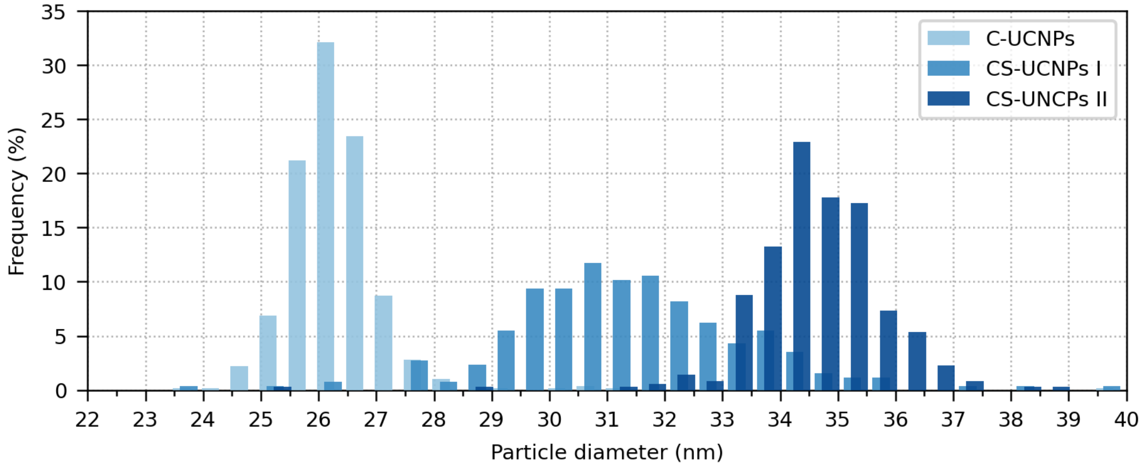

Figure S1. Particle size distributions obtained from the image analysis of TEM/BF micrographs for C-UCNPs (light blue), CS-UCNPs I (medium blue), and CS-UCNPs II nanoparticles (dark blue). At least 250 nanoparticles were measured for each sample.

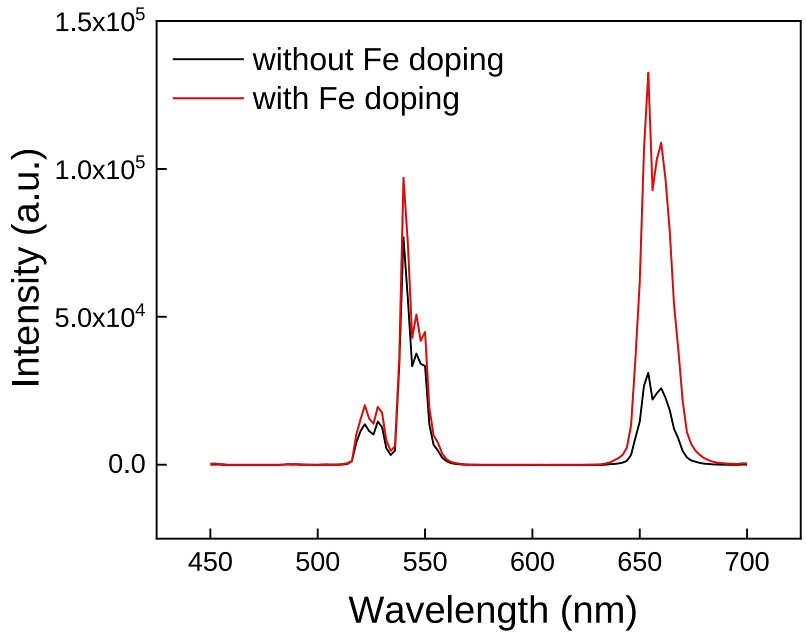

Figure S2. Upconversion photoluminescence spectra of C-UCNPs (2 mg/ml) codoped with Fe ions (0 or 10% charged in the reaction) after excitation at 980 nm (2.11 W/cm^2).

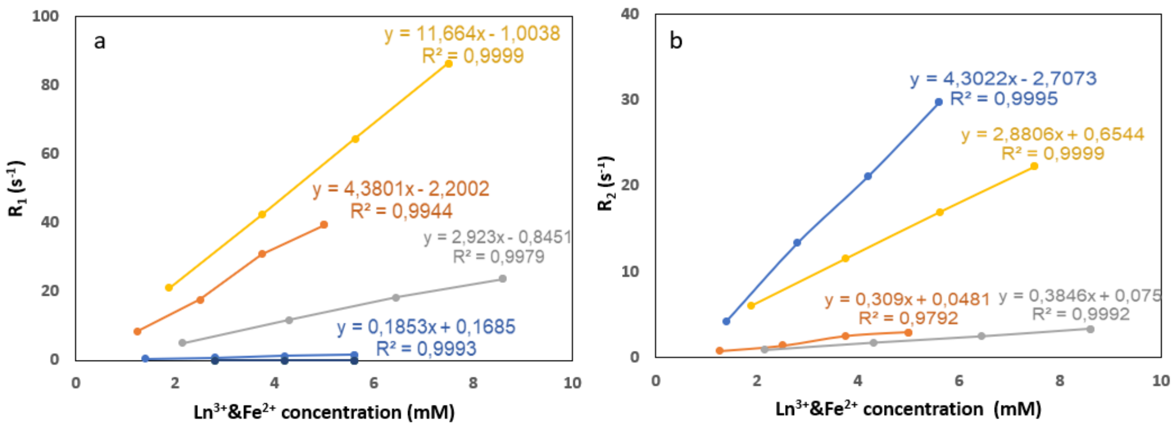

Figure S3. Relaxivity ($r_{1,2}$) of (blue) C-UCNPs, (red) CS-UCNPs I, (grey) CS-UCNPs II and (yellow) CS-UCNP@PMVEMA nanoparticles expressed as the slope of linear dependence of relaxation rate ($R_{1,2}$) on Me ion concentration. Data points represent mean values from three measurements (n = 3); SD values are reported in Table 2.

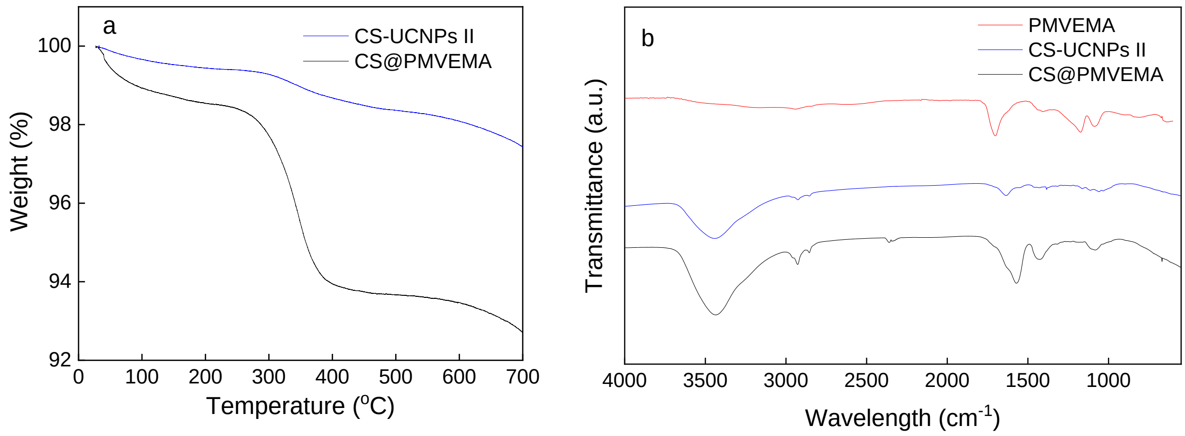

Figure S4. (a) TGA thermograms and (b) ATR-FTIR spectra of PMVEMA, neat CS-UCNPs II and CS-UCNP@PMVEMA nanoparticles.

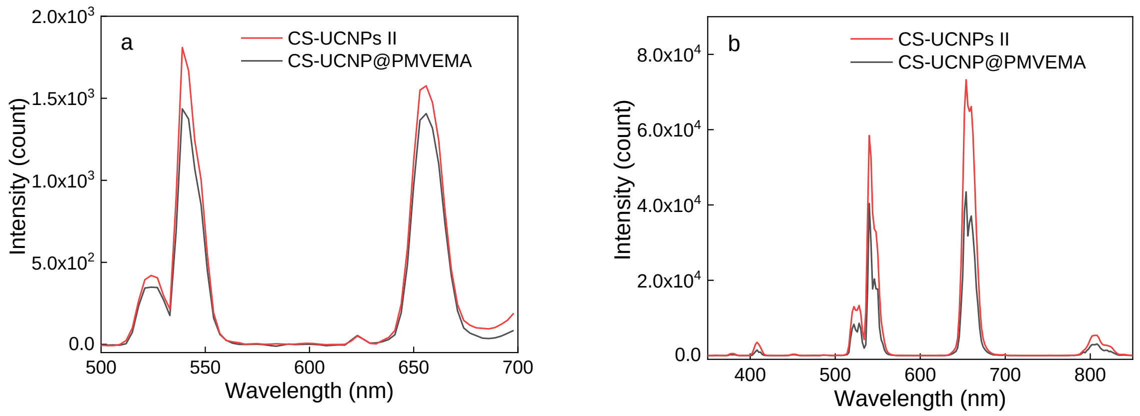

Figure S5. Upconversion emission spectra of neat CS-UCNPs II and CS-UCNP@PMVEMA particles in water (1 mg/ml) excited at (a) 808 and (b) 980 nm with laser power densities of 3 and 2.11 W/cm^2, respectively.
